# Robust Cooperative Fault-Tolerant Control for Uncertain Multi-Agent Systems Subject to Actuator Faults

**DOI:** 10.3390/s24082651

**Published:** 2024-04-21

**Authors:** Jiantao Shi, Xiang Chen, Shuangqing Xing, Anning Liu, Chuang Chen

**Affiliations:** College of Electrical Engineering and Control Science, Nanjing Tech University, Nanjing 211816, Chinachenxiang@njtech.edu.cn (X.C.); xingshuangqing@njtech.edu.cn (S.X.); liuanning@njtech.edu.cn (A.L.)

**Keywords:** multi-agent systems, robust fault estimation, cooperative fault-tolerance control, intermediate variable estimator

## Abstract

This article investigates the robust cooperative fault-tolerant control problem of multi-agent systems subject to mismatched uncertainties and actuator faults. During the design process of the intermediate variable estimator, there is no need to satisfy fault estimation matching conditions, and this overcomes a crucial constraint of traditional observers and estimators. The feedback term of the designed estimator contains the centralized estimation errors and the distributed estimation errors of the agent, and this further improves the design freedom of the proposed estimator. A novel fault-tolerant control protocol is designed based on the fault estimation information. In this work, the bounds of the fault and its derivatives are unknown, and the considered method is applicable to both directed and undirected multi-agent systems. Furthermore, the parameters of the estimator are determined through the resolution of a linear matrix inequality (LMI), which is decoupled by employing coordinate transformation and Schur decomposition. Lastly, a numerical simulation result is used to demonstrate the effectiveness of the proposed method.

## 1. Introduction

Spawned by the rapid advances in networked systems and distributed cooperation, there has been a flurry of activity on the topic of multi-agent systems (MASs). During the past few years, MASs have found extensive application in both industrial and military domains, such as multi-robot systems [1], satellite formation control [2], sensor networks [3], smart grids [4], flight control systems [5], etc.

As a type of interconnected system, MASs complete control tasks by exchanging information among neighboring agents. However, due to the system configuration and communication topology, a fault occurring in one agent can potentially propagate through the network, affect other agents, or even cause network disruption [6,7,8]. As a result, one specific agent fault could significantly impact MASs, potentially causing agent instability or even a system crash. Indeed, it is essential to highlight that faults may occur more frequently as the number of agents increases and as each agent expands in size and complexity. Furthermore, the security and reliability of MASs become increasingly crucial when they operate in complex and adverse environments.

To ensure the reliable operation of control systems, fault diagnosis has gained widespread attention in recent years and become a key focus of research. Fault diagnosis can be categorized into three main themes: fault detection (FD), fault isolation (FI), and fault estimation (FE) [9,10]. FD and FI primarily serve to detect and determine the location of faults, but they cannot obtain accurate fault information. In contrast, FE can provide detailed information about the shape and size of faults [11]. In general, the more fault information that can be obtained, the higher the fault-tolerant control performance [12]. Recently, a lot of fault estimation methods have been developed, including robust estimators, adaptive observers, sliding-mode controllers, and others [13,14]. However, there has been relatively limited research on fault estimation for MASs. For instance, ref. [15] addressed distributed fault estimation for interconnected systems with actuator faults. A novel distributed FD and FI method was proposed in [16] for heterogeneous MASs. To estimate sensor faults, a category of unknown input observers (UIO) was introduced in [17] by incorporating the system state and fault into a new extended vector. A robust fault estimation method using sliding-mode observers was derived in [18] for linear MASs subject to actuator faults. For the case of nonlinear MASs, an intermediate observer was introduced in [19] for estimating system states and actuator faults. Similar approaches were applied in [20] to construct nonlinear fault estimation observers for nonlinear MASs with sensor faults. In [21], timing FE observers were developed for interconnected systems with both multiplicative and additive actuator faults.

It is noteworthy that the majority of fault estimation methods in the aforementioned studies necessitate knowledge of the upper bound of the fault signal. However, since the bounds of fault information are mostly unknown for most control systems, these methods tend to be conservative in terms of fault estimation performance and applicability. Furthermore, in some existing works, the fault signal is designed as a constant, which is unrealistic since faults occurring in dynamic systems are inevitably influenced by environmental conditions and exhibit dynamic characteristics. Hence, these issues must be taken into consideration in the context of fault estimation for MASs.

Additionally, the fault estimation matching condition, which states that the rank of the product of the system output matrix and the fault distribution matrix should equal the rank of the system output matrix, is necessary for most existing fault estimation protocols. The aforementioned condition exhibits a higher level of conservatism when compared to the strict positivity assumption. In response to these challenges, a pioneering fault estimation method grounded in an intermediate observer was introduced in [22]. Notably, this approach dispenses with the necessity for both the fault estimation matching condition and the strictly positive real assumption.

Fault-tolerance control is the follow-up work of fault estimation. In the study of fault-tolerant control for MASs, there has been significant attention placed on observer-based active fault-tolerant control algorithms. In the process of designing fault-tolerant controllers, understanding the magnitude of the fault is crucial, and this information must be estimated by the constructed observers. For a class of MASs under a directed fixed topology, ref. [23] sidestepped the restriction of the requirement of zero initial conditions in most existing works and pioneered a novel delay-dependent fault-tolerant controller that solves the fault-tolerant constrained consensus problem of MASs in the presence of communication delays and actuator faults. Similarly, in order to sidestep the limitation of zero initial conditions in the H∞ control method, by using disturbance estimation information and a new performance function, ref. [24] designed a disturbance rejection adaptive fault-tolerant constrained consensus algorithm and solved the constraint consensus problem of perturbation-resilient adaptive fault-tolerant multi-agent systems under actuator failures. For linear and Lipschitz non-linear systems, an adaptive fault-tolerant controller utilizing the compensated actuator fault estimation information was proposed in [25] to mitigate adverse effects on consensus tracking. The problem of distributed fault-tolerant control for linear systems was explored in [26]. This approach utilized fuzzy logic systems to approximate unknown non-linear functions and implemented local observers to estimate the system states. In [27], a form of distributed adaptive fault-tolerant control strategy was proposed for linear MASs. This strategy aimed to alleviate the adverse impacts of actuator failures and losses in actuator effectiveness.

However, precise system models are necessary in the aforementioned works. Actually, model uncertainties are inevitable for practical systems and can stem from unknown internal or external noise, environmental influences, poor plant knowledge, and uncertain/slowly varying parameters. For multi-area power systems with actuator failures, ref. [28] considered the influence of uncertainty factors in the fault-tolerant controller and designed a fault-tolerant control scheme considering both multiplicative perturbation and additive perturbations. In [29], radial basis function neural networks (RBFNNs) were used to estimate dynamic uncertainties in the system model to ensure the accuracy of formation control. Similarly, the fuzzy logic method was used in [30] to address the problem of dynamic uncertainty estimation and further improve the accuracy of the proposed formation control algorithm. In [31], a distributed fault diagnosis method was created for a category of uncertain MASs with actuator faults. There was a need to design a state feedback controller ahead of the observer, suggesting the assumption that the full-dimensional state of the system can be measured. This does not align with the conditions observed in practical systems. A fault diagnosis algorithm based on an unknown input observer (UIO) was proposed in [32] for linear uncertain MASs. This work is only applicable to undirected MASs.

Unfortunately, most existing works have paid limited attention to fault estimation for MASs with uncertainties. Moreover, in the majority of these works, especially in the context of SMOs and UIOs, it is essential to fulfill the fault estimation matching condition and determine the bounds of the fault signal. Undoubtedly, these constraints greatly reduce the practicality and generality of traditional fault estimation and fault-tolerant control approaches.

Inspired by the above discussions, in this paper, the fault estimation and fault-tolerant control problem is addressed for a class of MASs with uncertainties. The innovations of this paper are as follows:

(1) A type of intermediate variable observer has been designed for each agent to estimate the fault and system state information. The fault estimation matching condition is not necessary for this approach, which is also applicable to time-varying faults.
(2) In the observer design process, both the centralized and distributed estimation errors of the agents are considered, which has the advantages of centralized and distributed structures and can enhance design flexibility and improve estimation performance.
(3) In the current work, it is not necessary to obtain the bounds of faults and their derivatives. Consequently, the distributed fault estimation method proposed in this paper has great generality and practicality.
(4) Compared with most existing results, this FE and FTC scheme is suitable for both directed and undirected MASs.

The structure of the paper is as follows. Section 2 gives some basic assumptions and describes the problem considered in this paper. Section 3 and Section 4 present the main theoretical results of this paper, including the construction of the intermediate variable observers and the convergence analysis. Section 5 presents a simulation example to demonstrate the effectiveness of the proposed method. Finally, the concluding remarks are given in Section 6.

*Notations:* In this article, $R^{n}$ is the *n*-dimensional Euclidean spaces and $I_{N}$ is an identity matrix of size N×N. For a vector $s\in R^{n}$, $∥s∥$ is the Euclidean norm. For a matrix D, $He\left[D\right]=D+D^{T}$, $\lambda_{max}\left(D\right)$ and $\lambda_{min}\left(D\right)$ represent the maximum and minimum eigenvalues of a matrix D, respectively.

## 2. Problem Statement

In this section, the problem formulation is presented. Consider the following linear MASs with unknown mismatched uncertainties

(1) $$\left\{\begin{array}{c} {\dot{x}}_{i}\left(t\right)=\left(A+\Delta A\left(t\right)\right)x_{i}\left(t\right)+Bu_{i}\left(t\right)+Ef_{i}\left(t\right)\\ y_{i}\left(t\right)=Cx_{i}\left(t\right) \end{array}\right.$$

where $x_{i}\left(t\right)\in R^{n}$ is the state information of agent *i*, and $u_{i}\left(t\right)\in R^{m}$ and $y_{i}\left(t\right)\in R^{q}$ are the control input and measured output of the system. $f_{i}\left(t\right)\in R^{r}$ is the fault signal and represents actuator faults when E=B. ΔA(t) is the perturbed matrix that satisfies ΔA(t)=MF(t)N, where $F^{T}\left(t\right)F\left(t\right)\leq I$. *A*, *B*, *E*, and *C* are the known constant matrices of the system, which have appropriate dimensions. Without loss of generality, in this paper, we assume that (A,C) is observable and (A,B) is stabilizable.

**Assumption** **1.** *In this paper, the fault $f_{i}\left(t\right)$ satisfies $∥\dot{f_{i}}\left(t\right)∥⩽\theta$ with θ⩾0.*

**Assumption** **2.** *$rank\left(E\right)=r$.*

**Assumption** **3.** *The following equation holds with respect to every complex number s*

$$Rank\left[\begin{array}{cc} A-sI & E\\ C & 0 \end{array}\right]=n+rank\left(E\right)$$

**Assumption** **4.** *$Rank\left(B,E\right)=rank\left(B\right)$.*

**Remark** **1.** *Assumption 1 gives the L-2 norm bounds of the fault and its derivative, which implies that the fault and its derivative are energy-bounded. This assumption is common in the field of FD and FTC [22,33] for estimating time-varying signals. In this paper, it is not necessary to know the specific information about the fault and its derivative bounds, i.e., θ is unknown, making the proposed approach more general than most traditional observers [13,14,17], in which the bounds of faults and their first derivatives must be known.*

**Remark** **2.** *It is important to note that Assumption 2 is more common than the fault estimation matching conditions proposed in many existing works. Assumption 3 is natural and pervasive in the majority of published articles that investigate fault detection, isolation, and estimation. This assumption implies that the system (A,C,E) has a constant amount of zeros in the left half-plane. Such a condition finds frequent application in the realms of system control and fault diagnosis.*

**Remark** **3.** *Assumption 4 is very common in the existing results of fault-tolerant control, implying that the fault is situated within the channel responsible for controlling the system’s input, and there is a likelihood that it can be mitigated through compensation via the control input.*

The following lemma is used for the subsequent work:

**Lemma** **1** ((Young’s Inequality) [19])**.** *For any two vectors, $a\in R^{n}$ and $b\in R^{n}$, we have*

$$a^{T}b⩽\frac{1}{p}\alpha^{p}{∥a∥}^{p}+\frac{1}{q}\alpha^{-q}{∥a∥}^{q}$$

*where* α>0, p>0, q>0, *and* pq=p+q.

In this paper, we design a fault estimator to acquire state information and fault information in real time and use the acquired information to design a fault-tolerant control protocol to compensate for the adverse effects of fault signals on MASs for the purpose of robust fault-tolerant cooperative control for MASs.

## 3. Intermediate Observer Design

In this subsection, an observer is designed for each agent *i*. To start with, the following intermediate variable $\xi_{i}\left(t\right)$ is denoted:

(2) $$\xi_{i}\left(t\right)=f_{i}\left(t\right)-\omega E^{T}x_{i}\left(t\right)$$

where ω is an intermediate constant selected based on experience.

From Equations (1) and (2), we have the dynamics of the intermediate variable:

(3) $$\begin{array}{cc} {\dot{\xi }}_{i}\left(t\right)= & {\dot{f}}_{i}\left(t\right)-\omega E^{T}\left(\left(A+\Delta A\right)x_{i}\left(t\right)+Bu_{i}\left(t\right)+E\xi_{i}\left(t\right)+\omega EE^{T}x_{i}\left(t\right)\right) \end{array}$$

Based on Equations (1)–(3), the following intermediate variable estimator is designed:

(4) $$\left\{\begin{array}{c} {\dot{\hat{x}}}_{i}\left(t\right)=A{\hat{x}}_{i}\left(t\right)+Bu_{i}\left(t\right)+E{\hat{f}}_{i}\left(t\right)+\rho_{1}L_{1}\Delta_{1i}+\rho_{2}L_{2}\Delta_{2i}\\ \begin{array}{c} {\dot{\hat{\xi }}}_{i}\left(t\right)=-\omega E^{T}E{\hat{\xi }}_{i}\left(t\right)\;-\;\omega E^{T}\left(A{\hat{x}}_{i}\left(t\right)\;+\;Bu_{i}\left(t\right)\;+\;\omega EE^{T}{\hat{x}}_{i}\left(t\right)\right) \end{array}\\ {\hat{f}}_{i}\left(t\right)={\hat{\xi }}_{i}\left(t\right)+\omega E^{T}{\hat{x}}_{i}\left(t\right)\\ {\hat{y}}_{i}\left(t\right)=C{\hat{x}}_{i}\left(t\right) \end{array}\right.$$

where ${\hat{x}}_{i}\left(t\right)$, ${\hat{\xi }}_{i}\left(t\right)$, ${\hat{f}}_{i}\left(t\right)$, and ${\hat{y}}_{i}\left(t\right)$ are the estimations of $x_{i}\left(t\right)$, $\xi_{i}\left(t\right)$, $f_{i}\left(t\right)$, and $y_{i}\left(t\right)$, respectively.

In addition, $\Delta_{1i}$ and $\Delta_{2i}$ are defined as

(5) $$\Delta_{1i}=y_{i}\left(t\right)-{\hat{y}}_{i}\left(t\right)$$

(6) $$\Delta_{2i}=\sum_{j=1}^{N}a_{ij}[(y_{i}\left(t\right)-{\hat{y}}_{i}\left(t\right))-\left(y_{j}\left(t\right)-{\hat{y}}_{j}\left(t\right)\right)]$$

which represent the centralized and distributed output estimation errors. $L_{1}\in R^{n}$ and $L_{2}\in R^{n}$ are the observer gain matrices, which are designed later. The non-negative constants $\rho_{1}$ and $\rho_{2}$ are the corresponding weight values that satisfy $0⩽\rho_{1}⩽1$, $0⩽\rho_{2}⩽1$, and $\rho_{1}+\rho_{2}=0$.

**Remark** **4.** *According to the designed intermediate variable estimator, it can be observed that both the centralized output estimation errors, $\Delta_{1i}$, and distributed output estimation errors, $\Delta_{2i}$, are taken into account. Here, $\rho_{1}$ and $\rho_{2}$ represent the weights assigned to the centralized and distributed output estimation errors, respectively. The magnitude of these values signifies the extent to which neighboring nodes influence the observer: a smaller $\rho_{1}$ (resulting in a larger $\rho_{2}$) amplifies the impact of neighboring nodes, whereas a larger $\rho_{1}$ (resulting in a smaller $\rho_{2}$) reduces the influence of neighboring nodes. In practice, the choice of $\rho_{1}$ and $\rho_{2}$ could increase the flexibility in designing the observers, and their specific selection should be tailored to the actual operating conditions.*

## 4. Estimation Error Analysis

Denote $e_{x_{i}}\left(t\right)=x_{i}\left(t\right)-{\hat{x}}_{i}\left(t\right)$, $e_{\xi_{i}}\left(t\right)=\xi_{i}\left(t\right)-{\hat{\xi }}_{i}\left(t\right)$, and $e_{f_{i}}\left(t\right)=f_{i}\left(t\right)-{\hat{f}}_{i}\left(t\right)$. Since $e_{f_{i}}\left(t\right)=e_{\xi_{i}}\left(t\right)+\omega E^{T}e_{x_{i}}\left(t\right)$, we can obtain the dynamics of the estimation error system as follows:

(7) $$\begin{array}{c} {\dot{e}}_{x_{i}}\left(t\right)=\left(A-\rho_{1}LC\right)e_{x_{i}}\left(t\right)+Ee_{\xi_{i}}\left(t\right)+\omega EE^{T}e_{x_{i}}\left(t\right)+\Delta Ax_{i}\left(t\right)-\rho_{2}L_{2}\Delta_{2i} \end{array}$$

(8) $$\begin{array}{c} {\dot{e}}_{\xi_{i}}\left(t\right)=\;-\;\omega E^{T}Ee_{\xi_{i}}\left(t\right)-\;\omega E^{T}Ae_{x_{i}}\left(t\right)\;-\;\omega^{2}EE^{T}Ee_{x_{i}}\left(t\right)-\omega E^{T}\Delta Ax_{i}\left(t\right)+{\dot{f}}_{i}\left(t\right) \end{array}$$

According to Assumption 4, there exists a matrix $B^{*}$ satisfying $(I-BB^{*})E=0$. Based on the fault estimation information, the control protocol proposed in this paper is presented as follows:

(9) $$u_{i}\left(t\right)=-K\hat{x_{i}}\left(t\right)-B^{*}E{\hat{f}}_{i}\left(t\right)$$

where *K* is designed to guarantee that A−BK is Hurwitz. Substituting Equation (9) into Equation (1), we can obtain

(10) $$\begin{array}{cc} {\dot{x}}_{i}\left(t\right)= & \left(A-BK\right)x_{i}\left(t\right)+\Delta Ax_{i}\left(t\right)+BKe_{x_{i}}\left(t\right)+Ee_{\xi_{i}}\left(t\right)+\omega EE^{T}e_{x_{i}}\left(t\right) \end{array}$$

From Equations (7), (8) and (10), the global error dynamic is

(11) $$\begin{array}{cc} \dot{x}\left(t\right)= & \left(I_{N}⊗\left(A-BK\right)\right)x\left(t\right)+\left(I_{N}⊗\Delta A\right)x\left(t\right)+\left(I_{N}⊗BK\right)e_{x}\left(t\right)\\ \; & +\left(I_{N}⊗E\right)e_{\xi }\left(t\right)+\left(I_{N}⊗\omega EE^{T}\right)e_{x}\left(t\right) \end{array}$$

(12) $$\begin{array}{cc} {\dot{e}}_{x}\left(t\right)= & \left(I_{N}⊗\left(A-\rho_{1}L_{1}C\right)\right)e_{x}\left(t\right)+\left(I_{N}⊗E\right)e_{\xi }\left(t\right)+\left(I_{N}⊗\omega EE^{T}\right)e_{x}\left(t\right)\\ \; & +\left(I_{N}⊗\Delta A\right)x\left(t\right)-\left(L⊗\rho_{2}L_{2}C\right)e_{x}\left(t\right) \end{array}$$

(13) $$\begin{array}{cc} {\dot{e}}_{\xi }\left(t\right)= & -\left(I_{N}⊗\omega E^{T}E\right)e_{\xi }\left(t\right)-\left(I_{N}⊗\omega E^{T}A\right)e_{x}\left(t\right)-\left(I_{N}⊗\omega^{2}EE^{T}E\right)e_{x}\left(t\right)\\ \; & -\left(I_{N}⊗\omega E^{T}\Delta A\right)x\left(t\right)+\dot{f}\left(t\right) \end{array}$$

where $x\left(t\right)={\left[x_{1}\left(t\right),x_{2}\left(t\right),\ldots ,x_{N}\left(t\right)\right]}^{T}$, $e_{x}\left(t\right)={\left[e_{x_{1}}\left(t\right),e_{x_{2}}\left(t\right),\ldots ,e_{x_{N}}\left(t\right)\right]}^{T}$, $e_{\xi }\left(t\right)={\left[e_{\xi_{1}}\;\left(t\right),e_{\xi_{2}}\;\left(t\right),\ldots ,e_{\xi_{N}}\;\left(t\right)\right]}^{T}$, $f\left(t\right)={\left[f_{1}\left(t\right),f_{2}\left(t\right),\ldots ,f_{N}\left(t\right)\right]}^{T}$.

**Theorem** **1.** *Under Assumptions 1–3, the intermediate variable estimator in Equation (4) guarantees that the global error dynamic in Equations (11)–(13) is uniformly ultimately bounded for the given intermediate constant ω>0, $\varepsilon_{i}>0$ (i=1,2,3,4), and there exists matrix $P_{j}>0$ (j=1,2,3), Q, and a constant δ>0 satisfying the following inequality:*

(14) $$\Pi^{i}=\left[\begin{array}{cc} {\tilde{\Pi }}_{11}^{i} & {\tilde{\Pi }}_{12}^{i}\\ {}* & {\tilde{\Pi }}_{22}^{i} \end{array}\right]<0,i=1,2,\ldots ,N$$

where ${\tilde{\Pi }}_{11}^{i}=\left[\begin{array}{ccc} \Pi_{11}^{i} & \Pi_{12}^{i} & \Pi_{13}^{i}\\ {}* & \Pi_{22}^{i} & \Pi_{23}^{i}\\ {}* & * & \Pi_{33}^{i} \end{array}\right]$, with $\Pi_{11}^{i}\;=\;He\left[P_{1}A\;-\;P_{1}BK\right]+\frac{1}{\varepsilon_{1}}N^{T}N+\frac{1}{\varepsilon_{2}}N^{T}N+\frac{1}{\varepsilon_{3}}N^{T}N$, $\Pi_{12}^{i}=P_{1}BK+\omega P_{1}EE^{T}$, $\Pi_{13}^{i}=P_{1}E$, $\Pi_{22}^{i}=He\left[\left(P_{2}A-\rho_{1}QC\right)+\left(\omega P_{2}EE^{T}\right)\right]+\delta \rho_{2}{\bar{\lambda }}_{i}C^{T}C$, $\Pi_{23}^{i}=P_{2}E-\omega A^{T}EP_{3}-\omega^{2}EE^{T}EP_{3}$, $\Pi_{33}^{i}=-He\left[\omega P_{3}E^{T}E\right]$, ${\tilde{\Pi }}_{22}^{i}=\mathrm{diag}\left\{-\frac{1}{\varepsilon_{1}},-\frac{1}{\varepsilon_{2}},-\frac{1}{\varepsilon_{3}},-\frac{1}{\varepsilon_{4}}\right\}$, ${\tilde{\Pi }}_{12}^{i}=\left[\begin{array}{cccc} P_{1}M & 0 & 0 & 0\\ 0 & P_{2}M & 0 & 0\\ 0 & 0 & \omega P_{3}E^{T}M & P_{3} \end{array}\right]$. The observer gain matrices can be designed as $L_{1}=P_{2}^{-1}Q$, $L_{2}=\delta P_{2}^{-1}C^{T}$.

**Proof of Theorem** **1.** Choose a Lyapunov function as follows:

(15) $$\begin{array}{cc} V(t)= & x^{T}\left(t\right)\left(I_{N}⊗P_{1}\right)x\left(t\right)+e_{x}^{T}\left(t\right)\left(I_{N}⊗P_{2}\right)e_{x}\left(t\right)+e_{\xi }^{T}\left(t\right)\left(I_{N}⊗P_{3}\right)e_{\xi }\left(t\right) \end{array}$$

According to Equations (11)–(13) and ΔA(t)=MF(t)N, one has

(16) $$\begin{array}{cc} \dot{V}\left(t\right)= & x^{T}\left(t\right)He\left[I_{N}⊗\left(P_{1}A-P_{1}BK\right)\right]x\left(t\right)+2x^{T}\left(I_{N}⊗P_{1}MF\left(t\right)N\right)x\left(t\right)\\ \; & +2x^{T}\left(I_{N}⊗P_{1}BK\right)e_{x}\left(t\right)+2x^{T}\left(t\right)\left(I_{N}⊗P_{1}E\right)e_{\xi }\left(t\right)\\ \; & +2x^{T}\left(t\right)\left(I_{N}⊗\omega P_{1}EE^{T}\right)e_{x}\left(t\right)-2e_{\xi }^{T}\left(t\right)\left(I_{N}⊗\omega P_{3}E^{T}A\right)e_{x}\left(t\right)\\ \; & +e_{x}^{T}\left(t\right)He\left[I_{N}⊗\left(P_{2}A-\rho_{1}P_{2}L_{1}C\right)\right]e_{x}\left(t\right)+e_{x}^{T}\left(t\right)He\left[L⊗(P_{2}A-\rho_{2}P_{2}L_{2}C)\right]e_{x}\left(t\right)\\ \; & +2e_{x}^{T}\left(t\right)\left(I_{N}⊗P_{2}E\right)e_{\xi }\left(t\right)+2e_{x}^{T}\left(t\right)\left(I_{N}⊗\omega P_{2}EE^{T}\right)e_{x}\left(t\right)\\ \; & -2\omega e_{\xi }^{T}\left(t\right)\left(I_{N}⊗P_{3}E^{T}MF\left(t\right)N\right)x\left(t\right)+2e_{x}^{T}\left(t\right)\left(I_{N}⊗P_{2}MF\left(t\right)N\right)x\left(t\right)\\ \; & -2e_{\xi }^{T}\left(t\right)\left(I_{N}⊗\omega P_{3}E^{T}E\right)e_{\xi }\left(t\right)-2e_{\xi }^{T}\left(t\right)\left(I_{N}⊗\omega^{2}P_{3}E^{T}EE^{T}\right)e_{x}\left(t\right)\\ \; & +2e_{\xi }^{T}\left(t\right)\left(I_{N}⊗P_{3}\right)\dot{f}\left(t\right) \end{array}$$

From Lemma 1 and $F\left(t\right)F^{T}\left(t\right)⩽I$, the following inequalities hold for the positive constants $\varepsilon_{1}>0$, $\varepsilon_{2}>0$, $\varepsilon_{3}>0$, and $\varepsilon_{4}>0$:

(17) $$\begin{array}{cc} 2x^{T}\left(t\right) & (I_{N}⊗P_{1}MF\left(t\right)N)x\left(t\right)\\ \leq & \varepsilon_{1}x^{T}\left(I_{N}⊗P_{1}M\right){\left(I_{N}⊗P_{1}M\right)}^{T}x\left(t\right)+\frac{1}{\varepsilon_{1}}x^{T}\left(I_{N}⊗N^{T}N\right)x\left(t\right)\\ 2e_{x}^{T}\left(t\right) & (I_{N}⊗P_{2}M_{1}F_{1}\left(t\right)N_{1})x\left(t\right)\\ \leq & \varepsilon_{2}e_{x}^{T}\left(t\right)\left(I_{N}⊗P_{2}M\right)\left(I_{N}⊗M^{T}P_{2}\right)e_{x}\left(t\right)+\frac{1}{\varepsilon_{2}}x^{T}\left(t\right)\left(I_{N}⊗N^{T}N\right)x\left(t\right)\\ -2e_{\xi }^{T}\left(t\right) & (I_{N}⊗\omega P_{3}E^{T}MF\left(t\right)N)x\left(t\right)\\ \leq & \varepsilon_{3}e_{\xi }^{T}\left(t\right)\left(I_{N}⊗\omega P_{3}E^{T}M\right)\left(I_{N}⊗\omega M^{T}EP_{3}\right)e_{\xi }\left(t\right)+\frac{1}{\varepsilon_{3}}x^{T}\left(t\right)\left(I_{N}⊗N^{T}N\right)x\left(t\right) \end{array}$$

Based on Assumption 1, it can be inferred that there exists a scalar $\theta_{N}>0$ such that the following inequality consistently holds:

(18) $$\begin{array}{cc} 2e_{\xi }^{T}\left(t\right)\left(I_{N}⊗P_{3}\right)\dot{f}\left(t\right)\leq & \frac{1}{\varepsilon_{4}}e_{\xi }^{T}\left(t\right)\left(I_{N}⊗P_{3}\right)\left(I_{N}⊗P_{3}\right)e_{\xi }\left(t\right)+\varepsilon_{4}\theta_{N} \end{array}$$

Furthermore, based on Equations (17) and (18), we have

(19) $$\begin{array}{cc} \dot{V}\left(t\right)\leq & x^{T}\left(t\right)\left[He[I_{N}⊗\left(P_{1}A-P_{1}BK\right)]\right]x\left(t\right)+\varepsilon_{1}x^{T}\left(t\right)\left(I_{N}⊗P_{1}MM^{T}P_{1}\right)x\left(t\right)\\ \; & +\frac{1}{\varepsilon_{1}}x^{T}\left(t\right)\left(I_{N}⊗N^{T}N\right)x\left(t\right)+2x^{T}\left(t\right)\left(I_{N}⊗\omega P_{1}EE^{T}\right)e_{x}\left(t\right)\\ \; & +2x^{T}\left(I_{N}⊗P_{1}BK\right)e_{x}\left(t\right)+2x^{T}\left(t\right)\left(I_{N}⊗P_{1}E\right)e_{\xi }\left(t\right)\\ \; & +e_{x}^{T}\left(t\right)\left[He[I_{N}⊗\left(P_{2}A-\rho_{1}QC\right)]\right]e_{x}\left(t\right)+e_{x}^{T}\left(t\right)\left[\left(L+L^{T}\right)⊗\left(\delta \rho_{2}C^{T}C\right)\right]e_{x}\left(t\right)\\ \; & +2e_{x}^{T}\left(t\right)\left(I_{N}⊗P_{2}E\right)e_{\xi }\left(t\right)+2e_{x}^{T}\left(t\right)\left(I_{N}⊗\omega P_{2}EE^{T}\right)e_{x}\left(t\right)\\ \; & +\varepsilon_{2}e_{x}^{T}\left(t\right)\left(I_{N}⊗P_{2}MM^{T}P_{2}\right)e_{x}\left(t\right)+\frac{1}{\varepsilon_{2}}x^{T}\left(t\right)\left(I_{N}⊗N^{T}N\right)x\left(t\right)\\ \; & -2e_{\xi }^{T}\left(t\right)\left(I_{N}⊗\omega P_{3}E^{T}E\right)e_{\xi }\left(t\right)-2e_{\xi }^{T}\left(t\right)\left(I_{N}⊗\omega P_{3}E^{T}A\right)e_{x}\left(t\right)\\ \; & -2e_{\xi }^{T}\left(t\right)\left(I_{N}⊗\omega^{2}P_{3}E^{T}EE^{T}\right)e_{x}\left(t\right)+\varepsilon_{3}e_{\xi }^{T}\left(t\right)\left(I_{N}⊗\omega^{2}P_{3}E^{T}MM^{T}EP_{3}\right)e_{\xi }\left(t\right)\\ \; & +\frac{1}{\varepsilon_{3}}x^{T}\left(t\right)\left(I_{N}⊗N^{T}N\right)x\left(t\right)+\frac{1}{\varepsilon_{4}}e_{\xi }^{T}\left(t\right)\left(I_{N}⊗P_{3}P_{3}\right)e_{\xi }\left(t\right)+\varepsilon_{4}\theta_{N} \end{array}$$

where $Q=P_{2}L_{1}$, $L_{2}=\delta P_{2}^{-1}C^{T}$.

Obviously, Equation (19) is equivalent to

(20) $$\dot{V}\left(t\right)\leq {\tilde{x}}^{T}\left(t\right)\Phi \tilde{x}\left(t\right)+\varepsilon_{4}\theta_{N}$$

where

(21) $$\tilde{x}\left(t\right)=\left[\begin{array}{c} x(t)\\ e_{x}\left(t\right)\\ e_{\xi }\left(t\right) \end{array}\right],\Phi =\left[\begin{array}{ccc} \Phi_{11} & \Phi_{12} & \Phi_{13}\\ {}* & \Phi_{22} & \Phi_{23}\\ {}* & * & \Phi_{33} \end{array}\right]$$

and $\Phi_{11}=He\left[I_{N}⊗\left(P_{1}A-P_{1}BK\right)\right]+\varepsilon_{1}\left(I_{N}⊗P_{1}MM^{T}P_{1}\right)+\frac{1}{\varepsilon_{1}}\left(I_{N}⊗N^{T}N\right)+\frac{1}{\varepsilon_{2}}\left(I_{N}⊗N^{T}N\right)+\frac{1}{\varepsilon_{3}}\left(I_{N}⊗N^{T}N\right)$, $\Phi_{12}=\left(I_{N}⊗P_{1}BK\right)\;+\;\left(I_{N}⊗\omega P_{1}EE^{T}\right)$, $\Phi_{13}=\left(I_{N}⊗P_{1}E\right)$, $\Phi_{22}=He\left[\left(I_{N}⊗\left(P_{2}A-\rho_{1}QC\right)\right)+\left(I_{N}⊗\omega P_{2}EE^{T}\right)\right]\;+\;\left(L+L^{T}\right)⊗\left(\delta \rho_{2}C^{T}C\right)\;+\;\left(I_{N}\;⊗P_{2}MM^{T}P_{2}\right)$, $\Phi_{23}=\left(I_{N}⊗P_{2}E\right)\;-\;\left(I_{N}\;⊗\omega A^{T}EP_{3}\right)\;-\;\left(I_{N}⊗\omega^{2}EE^{T}EP_{3}\right)$, $\Phi_{33}=-He\left[\left(I_{N}⊗\omega P_{3}E^{T}E\right)\right]+\varepsilon_{3}\left(I_{N}⊗\omega^{2}P_{3}E^{T}MM^{T}EP_{3}\right)+\varepsilon_{4}\left(I_{N}⊗P_{3}P_{3}\right)$.

From Equation (15), we can be obtain

(22) $$\begin{array}{cc} V(t) & \leq \max\left[\lambda_{\max}\left(P_{1}\right),\lambda_{\max}\left(P_{2}\right),\lambda_{\max}\left(P_{3}\right)\right]{\left(\|x\left(t\right)\right\|}^{2}+{∥e_{x}\left(t\right)∥}^{2}+{∥e_{\xi }\left(t\right)∥}^{2})\\ \; & =\max\left[\lambda_{\max}\left(P_{1}\right),\lambda_{\max}\left(P_{2}\right),\lambda_{\max}\left(P_{3}\right)\right]{∥e_{\tilde{x}}\left(t\right)∥}^{2} \end{array}$$

It follows that

(23) $${∥e_{\tilde{x}}\left(t\right)∥}^{2}\geq \frac{V(t)}{\max\left[\lambda_{\max}\left(P_{1}\right),\lambda_{\max}\left(P_{2}\right),\lambda_{\max}\left(P_{3}\right)\right]}$$

From Equation (20), we can obtain

(24) $$\dot{V}\left(t\right)\leq \lambda_{\max}\left(\Phi \right){∥e_{\tilde{x}}\left(t\right)∥}^{2}+\varepsilon_{4}\theta_{N}$$

It is obvious that $\lambda_{\max}\left(\Phi \right)<0$, and then we have

(25) $$\dot{V}\left(t\right)\leq \kappa V\left(t\right)+\alpha$$

where $\kappa =\frac{\lambda_{\max}\left(\Phi \right)}{\max\left[\lambda_{\max}\left(P_{1}\right)\lambda_{\max}\left(P_{2}\right),\lambda_{\max}\left(P_{3}\right)\right]}<0$, $\alpha =\varepsilon_{4}\theta_{N}$.

Denote a set Ω satisfying the following condition:

(26) $$\begin{array}{c} \Omega =\{\left(\;x\left(t\right),e_{x}\left(t\right),e_{\xi }\left(t\right)\;\right)|\lambda_{\min}\left(P_{1}\right)∥x\left(t\right){\|}^{2}\;\;+\;\lambda_{\min}\left(P_{2}\right)∥e_{x}\left(t\right){∥}^{2}\;\;+\lambda_{\min}\left(P_{3}\right)∥e_{\xi }\left(t\right){∥}^{2}\leq -\frac{\alpha }{\kappa }\} \end{array}$$

Let $\bar{\Omega }$ be the supplementary set of Ω, and then the following inequality holds:

(27) $$\begin{array}{cc} V(t) & \geq \lambda_{\min}(P_{1})∥{x\left(t\right)\|}^{2}+\lambda_{\min}\left(P_{2}\right){∥e_{x}\left(t\right)∥}^{2}+\lambda_{\min}\left(P_{3}\right){∥e_{\xi }\left(t\right)∥}^{2}\geq -\frac{\alpha }{\kappa } \end{array}$$

if $\left(x\left(t\right),e_{x}\left(t\right),e_{\xi }\left(t\right)\right)\in \bar{\Omega }$. From Equations (25) and (27), it is obvious that for $\left(x\left(t\right),e_{x}\left(t\right),e_{\xi }\left(t\right)\right)\in \bar{\Omega }$, we have

(28) $$\dot{V}\left(t\right)\leq 0$$

which means that $\left(x\left(t\right),e_{x}\left(t\right),e_{\xi }\left(t\right)\right)$ is uniformly ultimately bounded and converges to Ω exponentially with a rate greater than $e^{\alpha t}$ from the Lyapunov stability theory.

Obviously, the inequality Φ<0 is a preliminary condition to ensure the stability of the global error dynamic in Equations (11)–(13). However, it is not hard to find that Φ<0 is still a high-dimensional and nonlinear characteristic. Therefore, in order to further ensure the solvability of the inequality Φ<0, this condition needs to be further transformed. Note that the term $L+L^{T}$ in Equation (21) is real symmetric, which means that it must have *N* real eigenvalues. By spectral decomposition of the real symmetric matrix $L+L^{T}$, we obtain

(29) $$L+L^{T}=\bar{V}\bar{\Lambda }{\bar{V}}^{T}$$

where $\bar{V}$ is constructed from the eigenvectors of $L+L^{T}$, and $\bar{\Lambda }=\mathrm{diag}\left\{{\bar{\lambda }}_{1},{\bar{\lambda }}_{2},\ldots ,{\bar{\lambda }}_{N}\right\}$ and ${\bar{\lambda }}_{i}\left(i=1,2,\ldots ,N\right)$ are the corresponding eigenvalues of $L+L^{T}$. Certainly, the matrix $\bar{V}$ is orthogonal. Then, an orthogonal transformation matrix is defined as follows:

(30) $$H=\left[\begin{array}{ccc} {\bar{V}}^{T}⊗I_{n} & 0 & 0\\ 0 & {\bar{V}}^{T}⊗I_{n} & 0\\ 0 & 0 & {\bar{V}}^{T}⊗I_{r} \end{array}\right]$$

By pre-multiplying and post-multiplying Φ<0 with H and its transpose, we can obtain

(31) $$\tilde{\Phi }=\left[\begin{array}{ccc} \Phi_{11} & \Phi_{12} & \Phi_{13}\\ {}* & {\tilde{\Phi }}_{22} & \Phi_{23}\\ {}* & * & \Phi_{33} \end{array}\right]<0$$

where ${\tilde{\Phi }}_{22}=He\left[\left(I_{N}⊗\left(P_{2}A-\rho_{1}Q_{2}C\right)\right)+\left(I_{N}⊗\omega P_{2}EE^{T}\right)\right]+\bar{\Lambda }⊗\left(\delta \rho_{2}C^{T}C\right)+I_{N}⊗P_{2}M_{1}M_{1}^{T}P_{2}$, and it can be found that the other terms are the same as in Equation (21). Finally, based on the Schur complement lemma, it can be found that $\tilde{\Phi }<0$ is equivalent to Equation (14) for i=1,2,…,N. The proof of the theorem is complete. □

**Remark** **5.** *In fact, excessively high dimensionality can negatively affect the accuracy of the LMI solution and may even result in no feasible solution for the LMI. Therefore, decoupling and dimension reduction for the preliminary condition Φ<0 is very necessary.*

**Remark** **6.** *According to Equations (11)–(13), it can be found that there is a coupling between x(t), $e_{x}\left(t\right)$, and $e_{\xi }\left(t\right)$. In order to prove the convergence of the system state and the observation error at the same time, it is necessary to choose a Lyapunov function containing x(t), $e_{x}\left(t\right)$, and $e_{\xi }\left(t\right)$.*

**Remark** **7.** *In the proof of Theorem 1, by considering the term $e_{x}^{T}\left(t\right)\left[\left(L+L^{T}\right)⊗\left(\delta \rho_{2}C^{T}C\right)\right]e_{x}\left(t\right)$ in Equation (20), it is found that $L+L^{T}$ is a real symmetric matrix, that is, the Laplacian matrix L of MASs can be asymmetric. In other words, the topology of MASs can be either undirected or directed in this paper. Therefore, the method proposed in this paper has better universality and practicability compared with most existing results.*

**Remark** **8.** *In this paper, the system state and the estimation error are analyzed strictly for convergence, and the explicit boundary, $-\frac{\alpha }{\kappa }$, is obtained. Obviously, by adjusting the gain matrix K, parameters ω, and $\varepsilon_{j}\;\left(j=1,2,3,4\right)$, parameter α is sufficiently large, and thus a relatively small bound is obtained. In addition, it is not difficult to find the convergence rate of the system state, and the global error dynamic in Equations (11)–(13) can be quantified by directly selecting the proper matrix K and parameter ω. Once the matrix K and parameter ω are determined, the observer gain matrices, $L_{1}$ and $L_{2}$, can be obtained by solving the LMI in Equation (14). At the same time, the feasibility of the LMI in Equation (14) can be enhanced by adjusting parameters $\varepsilon_{j}\;\left(j=1,2,3,4\right)$. On the other hand, if the LMI in Equation (14) has a feasible solution, the estimation and control performance can be enhanced by adjusting the matrix K and parameter ω. Therefore, this paper makes full use of the design freedom of parameters to ensure feasibility and effectiveness.*

## 5. Numerical Simulation

In this section, a numerical simulation is used to demonstrate the effectiveness of the method proposed in this paper.

**Figure 1:**
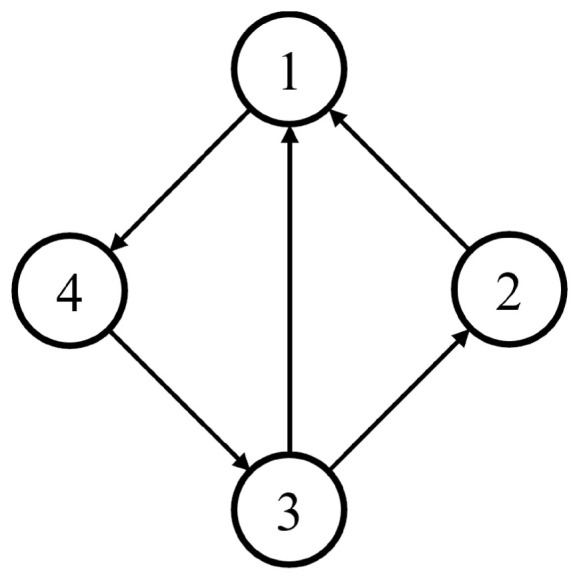
Topology of the MAS.

In this numerical simulation, a MAS with four agents is considered. The topology of the MAS is shown in Figure 1.

According to Figure 1, the adjacency matrix A and the Laplacian matrix L of the MAS are as follows.

$$A=\left[\begin{array}{cccc} 0 & 0 & 0 & 1\\ 1 & 0 & 0 & 0\\ 1 & 1 & 0 & 0\\ 0 & 0 & 1 & 0 \end{array}\right],L=\left[\begin{array}{cccc} 1 & 0 & 0 & -1\\ -1 & 1 & 0 & 0\\ -1 & -1 & 2 & 0\\ 0 & 0 & -1 & 1 \end{array}\right]$$

Obviously, since we consider a directed graph, the Laplacian matrix L is not symmetric.

In this paper, the actuator fault is considered. The system parameters of the *i*th (i=1,2,3,4) agent are given as

$$A=\left[\begin{array}{ccc} -1 & -2 & -3\\ -2 & -2 & 1\\ -3 & 1 & 1 \end{array}\right],B=E=\left[\begin{array}{c} 0\\ 0\\ -2 \end{array}\right],C=\left[\begin{array}{ccc} 1 & 0 & 0\\ 0 & 1 & 0 \end{array}\right]$$

$$M=\left[\begin{array}{ccc} 1 & 0 & 0\\ 0 & 1 & 0\\ 0 & 0 & 1 \end{array}\right],N=\left[\begin{array}{ccc} 0.5 & 0 & 0\\ 0 & 0.2 & 0\\ 0 & 0 & 0.1 \end{array}\right],F\left(t\right)=\sin\left(t\right)$$

Obviously, it is not hard to find that rank(CE)≠rank(E), that is, the fault estimation matching condition is not satisfied. In order to make the matrices (A−BK) Hurwitz, the gain matrix *K* is chosen as $K=\left[\begin{array}{ccc} 7.1153 & -4.6538 & -7.50 \end{array}\right]$.

Without loss of generality, we make the following assumptions about the fault of each of the four agents, respectively:

$$f_{1}\left(t\right)=\left\{\begin{array}{cc} 0 & 0s⩽t⩽10s\\ \sin\left(0.5t\right)+\cos\left(0.2t\right) & 10s<t⩽100s \end{array}\right.$$

$$f_{2}\left(t\right)=\left\{\begin{array}{cc} 0 & 0s⩽t⩽20s\\ 0.1t-2 & 20s<t⩽60s\\ 0.1t-5 & 60s<t⩽100s \end{array}\right.$$

$$f_{3}\left(t\right)=\left\{\begin{array}{cc} 0 & 0s⩽t⩽30s\\ 2\left(1-e^{-\left(t-30\right)}\right) & 30s<t⩽60s\\ 1-2\left(1-e^{-\left(t-60\right)}\right) & 60s<t⩽100s \end{array}\right.$$

$$f_{4}\left(t\right)=\begin{array}{cc} 0 & 0s⩽t⩽100s \end{array}$$

Based on the fault estimator given in the previous section, we choose the weight values as $\rho_{1}=0.3$ and $\rho_{2}=0.7$, respectively. The intermediate constant ω in Equation (2) is selected as ω=0.2. Based on Theorem 1, the gain matrices of the intermediate variable estimator in Equation (4) can be expressed as follows:

$$L_{1}=\left[\begin{array}{cc} 17.5310 & -3.9315\\ -32.1705 & 2.0079\\ 69.3836 & -66.3622 \end{array}\right],L_{2}=\left[\begin{array}{cc} -0.0261 & -0.3609\\ 0.0088 & 0.4167\\ -0.9831 & -2.3536 \end{array}\right]$$

**Figure 2:**
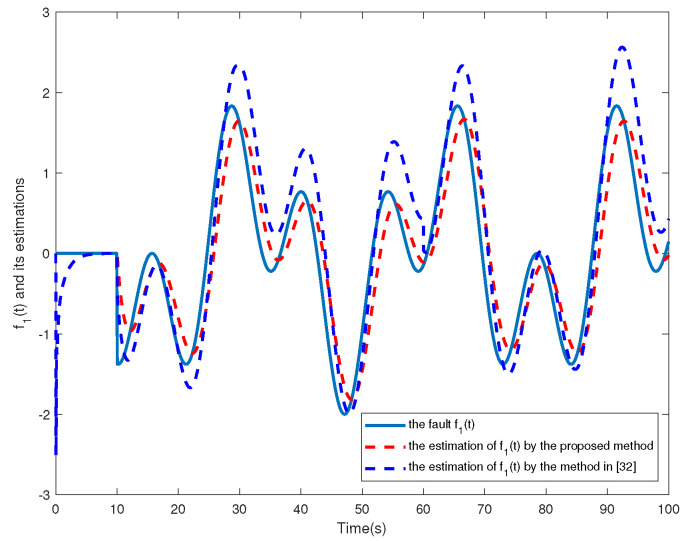
$f_{1}\left(t\right)$ and its estimations in agent 1.

**Figure 3:**
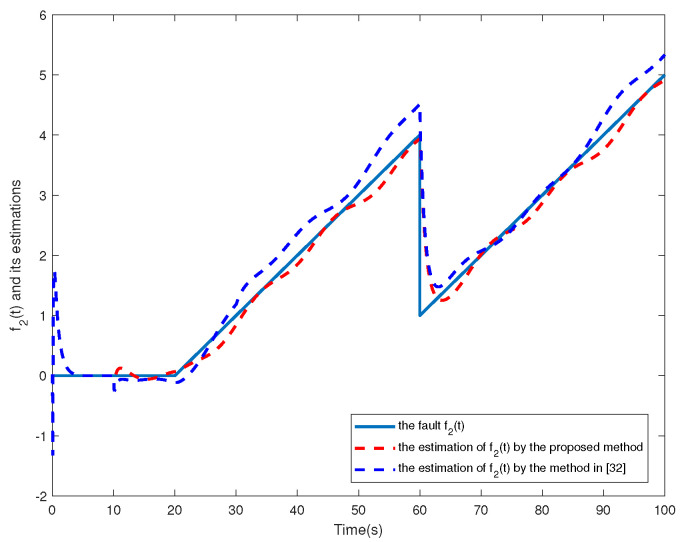
$f_{2}\left(t\right)$ and its estimations in agent 2.

**Figure 4:**
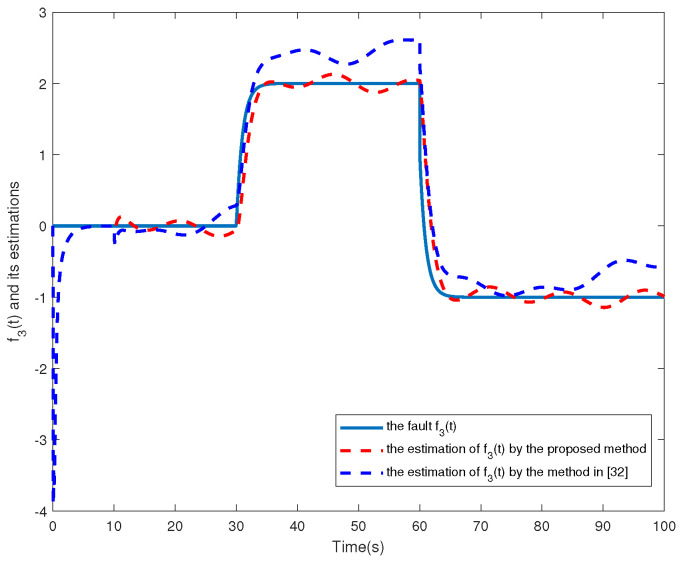
$f_{3}\left(t\right)$ and its estimations in agent 3.

**Figure 5:**
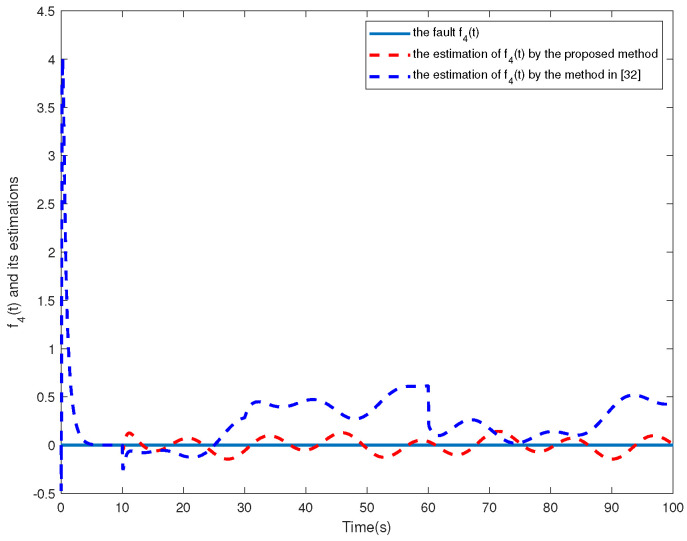
$f_{4}\left(t\right)$ and its estimations in agent 4.

**Figure 6:**
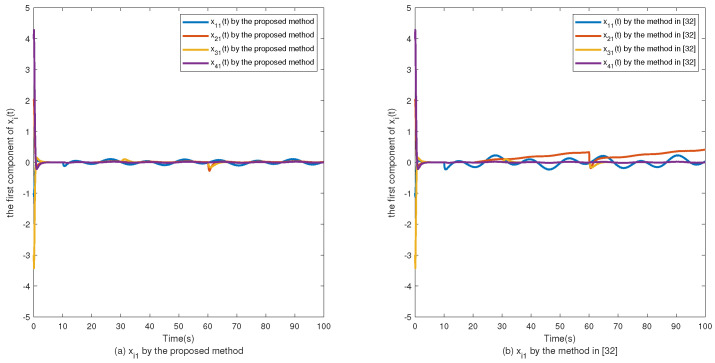
The first component of $x_{i}\left(t\right)$.

**Figure 7:**
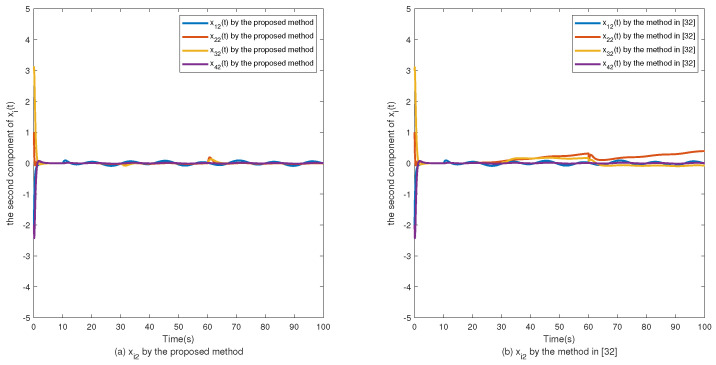
The second component of $x_{i}\left(t\right)$.

**Figure 8:**
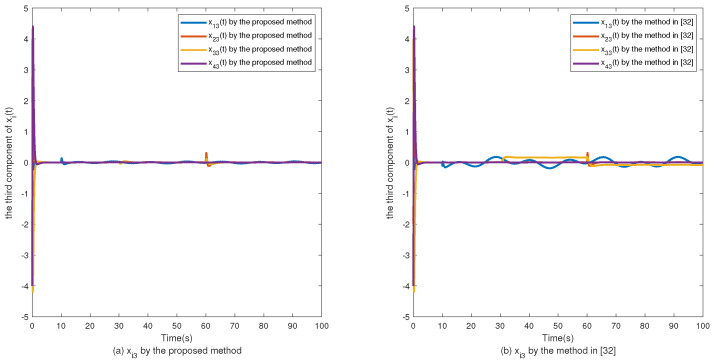
The third component of $x_{i}\left(t\right)$.

The simulation results are shown in Figure 2, Figure 3, Figure 4, Figure 5, Figure 6, Figure 7 and Figure 8. Figure 2, Figure 3, Figure 4 and Figure 5 show the effectiveness of fault estimation for the actuators of the four agents, respectively, where the red dashed line represents the result of the proposed method and the blue dashed line represents the result of the method proposed in [32]. As can be seen in Figure 2, Figure 3, Figure 4 and Figure 5, the fault estimation results of all agents are more satisfactory. Figure 6, Figure 7 and Figure 8 validate the efficacy of the fault-tolerant control protocol presented in this paper, and the results are better than those of the method proposed in [32]. Obviously, the state component of each agent converges to a small enough range under the designed fault-tolerant control protocol, and the influence of the actuator fault on the convergence process is greatly reduced. In addition, it is found that the fault estimation matching condition of fault estimation is not satisfied, and the bounds of the fault and its derivatives are not required. Therefore, the distributed fault estimation method based on the intermediate variable observer and the fault-tolerant control protocol based on fault estimation proposed in this paper are validated on a MAS with uncertainty.

## 6. Conclusions

In this paper, a novel robust distributed cooperative fault-tolerant control protocol is designed for a class of MASs with uncertainty and actuator faults. Unlike most existing approaches, in our method, the fault estimation matching condition is not necessary, and the bounds of the fault and its derivative are unknown. By introducing an intermediate variable and using both the centralized estimation errors and distributed estimation errors, the actuator fault is estimated, providing the basis for the fault-tolerance control scheme. Coordinate transformation and Schur decomposition are used to further reduce and decouple the LMI with high dimension and interference coupling, based on which the gain matrix of the estimator can be guaranteed. In future work, distributed cooperative fault-tolerant control for MASs with uncertainties in output and control input channels will be further considered. 

## Data Availability

Data are contained within the article.
